# Annotations

**Published:** 1900-10-20

**Authors:** 


### Annotations.

### Notification on Suspicion.

As we pointed out last week, it is often extremely difficult in the early stages of plague to come to a definite diagnosis as to the nature of the disease. Yet under the present system, until a diagnosis can be made, notification does not take place, and it seems not impossible that the widespread interest just now shown in all that concerns plague and its prevention may lead to some alteration in the wording of the Notification Act, so as to enable the authorities to obtain information as to the existence of infectious disease at an earlier stage than is provided for by the Act as it now stands. The words of the Act are so framed that there is no compulsion upon anyone, whether medical practitioner, householder, or relative, to notify the existence of any infectious disease until he or she "becomes aware that the patient is suffering from an infectious disease to which this Act applies," and the result of this unfortunate arrangement has been that endless delays have taken place in notifying such diseases. It must be borne in mind that in sending in a report of a case occurring in his private practice, a medical man is doing a thing which would be utterly unjustifiable but for the statute by which the duty of reporting is imposed upon him. It is only the obligation imposed upon him by law that protects the medical man from the consequences of what would otherwise be uncalled-for intermeddling; and it has been very strongly felt by medical men that they would have no protection if they were to report diseases not included in the schedule of the Act, or if they were to report any case on mere suspicion, before "becoming aware" of the nature of the disease. Thus it has happened that sometimes practitioners have not reported cases of typhoid fever until, by running through its full course, all doubt as to the nature of the malady had been removed, and have not reported cases of scarlet fever until peeling had taken place. These are extreme instances, but again and again it has happened that valuable time has been lost while the doctor has waited to become aware, not that the disease was an infectious one, but that, according to the wording of the Act, it was a "disease to which this Act applies." Such a condition of affairs is bad enough in regard to scarlet fever and diphtheria—so bad that it has long been held by many medical officers of health that all sore throats among children ought to be notified. But with plague it is still worse. On the one hand we may have cases of plague mimicking pneumonia or septicæmia, or being in turn imitated by such diseases, as in a case reported from Glasgow last week, in which a septic sore throat with a chain of enlarged glands and acute septicæmia accurately simulated a case of plague; and, on the other hand, we may have patients only slightly ill, suffering from the so-called ambulant form of plague, in which the real nature of the disease may be merely a matter of suspicion. If, then, notification is to be really useful, it must be given long before the doctor is "aware" of the nature of the malady. Sir W. T. Gairdner has recently drawn attention to this weak spot in the Notification Act, and has urged that this Act ought to have been so constructed that every case—not only of fully formed infectious disease, but every case in which a reasonable presumption of infection exists—should be at once reported, leaving the diagnosis in some cases to follow the notification. Some alteration of the wording in this sense will have to be made if early notification is to be obtained, and if the obligation to notify, which already lies upon the head of the family, the relatives present, and even upon the householder, is to be enforced as it should be. No doubt the result of such a change would be to considerably increase the amount paid for notification, but that would be a trifle. The point is that at present much of the money paid for notification is being thrown away. To pay for the notification of what everyone already knows is not good business. What the sanitary authorities really require is notification in the stage, not of knowledge but of suspicion, and for that they must be prepared to pay.

### Open-air Treatment for Consumptive Paupers.

Attention is again drawn to the important question of the open-air treatment of consumption by the proposal now made that the guardians of various London parishes should combine to make some provision for the treatment of the consumptive poor of London in accordance with this method. In working out the details of the proposed scheme, it would be well that those concerned should, at as early a stage of their proceedings as possible, take care to cut themselves adrift from the hampering and restrictive influence of the unfortunate phrase "open-air treatment" which is now so commonly used to indicate what should more properly be called the hygienic treatment of consumption, for our paupers will find themselves in but poor plight if their guardians should get the idea that in giving them "open air" they are offering them all that is necessary for the cure of their disease. That open air does good no one will deny, and that part of that good is derived from what may be termed its direct effect is very probable, but the fact remains that much of the benefit derived from living in the open is the result of its influence upon assimilation. It has always been observed that the condition of the digestive organs was the key to prognosis in phthisis, and practical men have found it more to the point to watch the appetite and the weight of patients affected with that disease than to pay too much attention to the physical signs within the chest, or to the number of bacilli in the expectoration. It is then to the power which life in the open seems to give of assimilating large quantities of food that much of the benefit of open-air treatment must be ascribed, and this must be reckoned with in the estimates.

Again, all those who have studied the hygienic treatment of consumption agree that much of the admitted success which has been met with is the result of constant and painstaking supervision by skilled medical men, and here again we would point out that the number of patients over whom any one medical man can exercise such supervision is but limited. Those then who enter upon a scheme for providing the consumptive poor of London with hygienic treatment, must recognise that anything like cheeseparing will spell failure. Not merely fresh air, but a most liberal dietary, a most constant medical superintendence, and a somewhat expensive domestic management, form essential parts of hygienic treatment during the more acute phases of the disease, while after this has been passed through, there comes the real difficulty which will still face the guardians—namely, what to do with the man whose disease is checked—is perhaps for the time being cured—but who, if he returns to his old surroundings, is practically certain to be attacked again by his old malady. The treatment within the sanatorium is but the fringe of a very great subject.

### The Late Sir Henry Acland.

Through the death of Sir Henry Acland Oxford loses one of the few who remain of that remarkable circle of interesting men amongst whom were numbered Pusey, Jowett, and Liddon. Sir Henry Acland was Regius Professor of Medicine in the University of Oxford for nearly 50 years; but it was through his personality, and not through the importance of his post, that he became one of the most striking individuals in the University. Nevertheless through his work he left an indelible record. He may be regarded as the founder of the Oxford Museum, which has become the centre of all the medical and scientific work and teaching in the University, and he was a consistent supporter of all movements to establish the Oxford School of Medicine on the highest basis. He was an enthusiastic upholder of the principles of sanitation, and carried his theories into practice at a village near Oxford, which he planned upon a model system. He was a Fellow of the Royal Society, the recipient of several honorary degrees from other universities, and was honorary member of numerous foreign societies. He held the post of President of the General Medical Council, and was Honorary Physician to the Pri c[illegible] of Wales and to the Duke of Albany during his residence in Oxford. Sir Henry Acland's interests extended far beyond the sphere of medicine, for, possessing as he did both taste and ability in art and literature, his interests in the University life were far-reaching. His courtesy and kindness to the younger men around him, as well as to all with whom he came in contact, have left an impression never to be forgotten. Others may fill the post he held in the University, but none can occupy the position he created through his strong individuality.

### The College Woman's Influence on the Race.

It certainly is hard upon the cultured person not to be able to transmit the intellectual polish acquired with such industry to coming generations. It is Dame Nature that is at the bottom of the mischief, for she not only muddles up in our children the tendencies derived from our own refinement with those passed on from hundreds of possibly quite vulgar ancestors, but, worse still, she demands that two people shall be engaged in the making of every child, so that the little brat's hereditary instincts are doubly mixed. In fact, it would seem as if all Nature's efforts were devoted to perpetuating the breed of rough-and-tumble average man—which is hard upon intellectual "freaks." The only hope for intellect, then, would seem to lie in taking a leaf out of Nature's book and bringing brainy men and brainy women together. Then, at last, the intermingled product might tell upon the race. Unfortunately, man in his matrimonial arrangements is apt to be an opportunist, and the brainy woman is not always at hand just when she is wanted. At last, however, the salvation of the race is found in the "college woman," for her uprising gives an opportunity of brain meeting brain quite in accordance with Nature's laws. Mrs. Mary Roberts Smith, a professor of sociology in an American university, has lately been collecting some comparative statistics as to college and non-college women in relation to marriage, child-birth, and health, the two groups being each other's sisters, cousins, or friends so as to ensure as far as possible similar social environment. These statistics are published in the last bulletin of the American Statistical Association, and they go to show that—

1. The college women marry two years later than the non-college women.

2. The college women have a considerably higher percentage of male children than the non-college women.

3. There is no measurable difference between the two classes in regard to health before or after marriage, or in regard to the health or mortality of their children.

4. Three-fourths of the college women married college men, while only one-half of the non-college women married college men. In other words, co-education promotes matrimony within the group of co-educated people.

5. Sixty-five per cent. of the college women, as compared with thirty-seven per cent. of the non-college women, married professional men.

All of which, if the figures are worth anything, is interesting in its way, showing that intellectual culture does not alter man's natural tendency to choose his wife from among those with whom he comes in daily contact, and suggesting that if we want to breed brains we must scatter brainy women in the path of brainy men. And much good may such brains do their owners. A good digestion might perhaps be a happier inheritance.

